# Preparation and Structural Analysis of Nano-Silver Loaded Poly(styrene-*co*-acrylic acid) Core-Shell Nanospheres with Defined Shape and Composition

**DOI:** 10.3390/nano7090234

**Published:** 2017-08-23

**Authors:** Jin Zhang, Xiaoyu Zhao, Yanfei Wang, Liang Zhu, Libin Yang, Gang Li, Zuoliang Sha

**Affiliations:** 1 Tianjin Key Laboratory of Marine Resources and Chemistry, College of Chemical Engineering and Materials Science, Tianjin University of Science and Technology, Tianjin 300457, China; zhangjin@mail.tust.edu.cn (J.Z.); zhuliang@tust.edu.cn (L.Z.); yanglibin@tust.edu.cn (L.Y.); zsha@tust.edu.cn (Z.S.); 2 State Key Laboratory of Environmental Chemistry and Ecotoxicology, Research Center for Eco-Environmental Sciences, Chinese Academy of Sciences, Beijing 100085, China; gangli@rcees.ac.cn

**Keywords:** core-shell structure, composite nanospheres, polymers, synthesis, structural analysis

## Abstract

A systematic study for the preparation and structural analysis of poly(styrene-*co*-acrylic acid) composite nanospheres (PSA) and silver nanoparticles loaded poly(styrene-*co*-acrylic acid) composite nanospheres (nAg@PSA) is reported. Poly(styrene-*co*-acrylic acid) nanospheres were synthesized by soap-free emulsion polymerization of styrene (St) and acrylic acid (AA) in water. Ag nanoparticles (Ag-NPs) were well-dispersed on the surfaces of poly(styrene-*co*-acrylic acid) composite nanospheres by in situ chemical reduction of AgNO_3_ using NaBH_4_ as a reducing agent in water. The particle size of PSA nanospheres was uniform. The surfaces of PSA nanospheres were distributed by highly uniform half-sphere arrays. Those half-sphere protruded more with the increase of the feeding amount of AA or the feed ratios of AA and St. The carboxyl groups content of nanospheres was directly proportional to the nanosphere surface area. This relationship and X-ray photoelectron spectroscopy and transmission electron microscopy images of the PSA nanospheres indicate that the acrylic acid was mainly distributed on the surface of the polystyrene spheres with unnegligible thickness. The number of Ag-NPs depends on immobilized carboxyl groups on the surface of PSA, according to thermogravimetry, ultraviolet-visible, X-ray diffraction and transmission electron microscopy results.

## 1. Introduction

Composite microspheres consisting of dielectric polymer core and metallic shells have become a subject of intense interest in various fields during past decades. The interest in these microspheres stems from their unique catalytic and optical properties, which are different from their bulk counterparts and hence, lead to widespread applications in catalysis, photonics, and so on [1,2,3,4,5,6,7,8,9,10,11,12,13,14,15]. Core-shell composite microspheres can effectively prevent metal nanoparticles from accumulation and improve catalytic stabilization [7]. By variation of the shell thickness and the core radius it is possible to adjust the plasmon resonance of the metallic shell particles over the whole visible and infrared region of the spectrum [5]. Because of their monodispersity and tunable optical properties, these microspheres may be used as building blocks for various applications such as for metallodielectric photonic crystals. A great deal of effort has been focused on preparing core-shell composite nanospheres with noble metallic shells, because they exhibit novel optical and catalytic properties and, in particular, surface enhanced Raman spectroscopy [16,17,18]. Jiang et al. [7] prepared SiO_2_/Ag core-shell particles and investigated the catalytic properties of silver nanoparticles supported on silica spheres in a colloidal solution. It was experimentally demonstrated that metal nanoparticles have high catalytic activities. Hu et al. [19] synthesized Ag-coated Fe_3_O_4_@SiO_2_ three-ply composite microspheres with surface-enhanced Raman scattering (SERS) properties. The Ag-coated Fe_3_O_4_@SiO_2_ microspheres were applied to detecting melamine, and strong SERS signals were obtained with melamine concentration of 1 × 10^−6^ M.

Various synthesis methods have been developed, such as self-assembly [20], seeding plating method [16], in situ reduction deposition method [21], and so on. Cassagnneau et al. [20] proposed the layer-by-layer (LbL) self-assembly method to form poly(styrene) core/silver nanoparticles shell composite materials. Zhang et al. [16] explored the seeding plating approach, based on electrostatic attraction, to prepare a complete silver shell with controlled thickness on silica colloids. Complete and continuous metallic shells can be formed by repeating the reduction and growth process. In the self-assembly and seeding plating methods, they have many characteristics: many preparation steps, long time, complexity of operation and so on. Hence, it is difficult to control the repeatability of the experiment. Recently, an in situ reduction deposition method has been reported. Because this method for preparing dielectric core/metallic shell composite materials is simple, reproducible and well-controlled, it has attracted much attention and is widely used in the preparation of core-shell structure composite microspheres.

Herein, we report the systematic study of poly(styrene-*co*-acrylic acid) composite nanospheres (PSA) and the deposition of uniform Ag-NPs shells on poly(styrene-*co*-acrylic acid) nanospheres by in situ reduction of AgNO_3_ using NaBH_4_ as a reducing agent in water. The key factors on structure and morphology of PSA nanospheres and silver nanoparticles loaded poly(styrene-*co*-acrylic acid) composite nanospheres (nAg@PSA) composite nanospheres were studied. Specifically, the relationship between the amount of carboxyl per nanosphere and surface geometry of nanospheres was revealed by titration combined with scanning electron microscopy (SEM) images. The surface geometry of PSA nanospheres was related to the amount of carboxyl groups per nanosphere. The titration, X-ray photoelectron spectroscopy (XPS) and transmission electron microscopy (TEM) results indicate that the carboxyl groups were mainly distributed on the surface of poly(styrene-*co*-acrylic acid) composite nanospheres comprising styrene core covered with acrylic acid shell. Ag nanoparticles were immobilized onto the PSA nanospheres with linear relationship with carboxy amount. This work may rise wide interest on structure and composition controllable preparation of core-shell structure microsphere for application in catalysis, antibacterial, substrate materials for surface enhanced Raman spectroscopy, and so on.

## 2. Experimental Section

### 2.1. Materials

Styrene (St) was supplied by Tokyo Chemical Industry Co., Ltd (Tokyo, Japan). Acrylic acid (AA), potassium persulfate (KPS), sodium borohydride (NaBH_4_), silver nitrate (AgNO_3_) and sodium hydroxide were purchased from Tianjin Chemical Reagent Co., Ltd (Tianjin, China). All chemicals were of analytical grade, and were used as received. Distilled water was used in all the experiments. All glassware used in the experiments was cleaned with distilled water before use.

### 2.2. Synthesis of Poly(styrene-co-acrylic acid) (PSA) Composite Nanospheres

**Figure 1:**
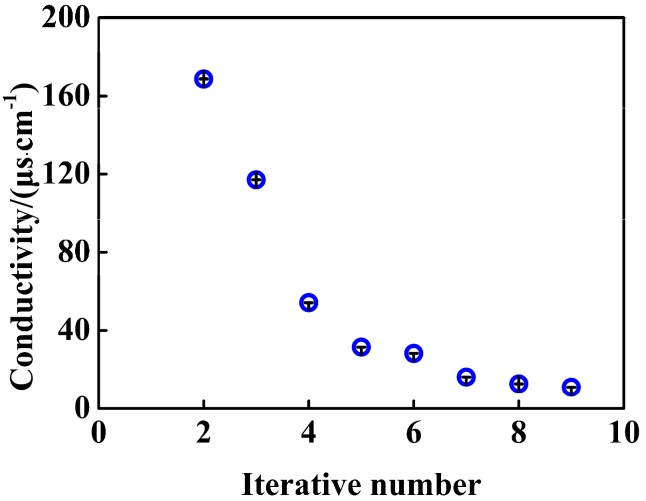
Dependence of conductivity of the PSA supernatant on the number of dispersion–centrifugation processes, a case of PSA0.

Poly(styrene-*co*-acrylic acid) (PSA) composite nanospheres were prepared by soap-free emulsion polymerization of styrene (St) and acrylic acid (AA) in water according to the method as described in the literature [22,23,24]. Briefly, a certain amount of AA and 130 mL of H_2_O were initially charged into a double walled container, which allowed ethylene glycol/water mixture to be circulated to keep the temperature constant. After the feeding AA was fully dissolved, the amount of styrene was added. The solution was purged with nitrogen to remove oxygen for 30 min and then heated to 75 °C under stirring. A solution of KPS dissolved in 20 mL of H_2_O was injected into the reaction mixture to initiate the polymerization, and reaction was maintained at 75 °C for 12 h. The stirrer speed, dosage of styrene, acrylic acid and potassium persulfate were shown in Table 1. As prepared PSA nanospheres were dispersed in water. The aqueous dispersion contained impurities caused by incomplete reaction before purification. Impurity of ions in the suspensions might enhance conduction unexpectedly by promoting dissociation of –COOH of PSA nanospheres. Conductance of a supernatant of the suspension is a measure of impurity of ion. The PSA suspension was centrifuged for 40 min at the rate of 12,000 r·min^−1^ and the precipitate was dispersed in 30 mL of distilled water. By determining the variation of the conductivity of the supernatant with the number of iterations of the dispersion-centrifugation processes, the purity of final PSA aqueous dispersion can be confirmed, as was shown in Figure 1. Values of conductivity of the PSA suspension decreased with an increase of the iterative steps of centrifugation-redispersion, and tended towards stability at the seventh. Consequently, ionic impurities were removed basically, so that the dissociation of –COOH of PSA maintains stable.

### 2.3. Preparation of nAg@PSA Composite Nanospheres

A typical procedure for fabricating nAg@PSA nanocomposites is described as follows: 200 mL of aqueous PSA dispersion (0.3 mg·mL^−1^) was mixed with 10 mL of 10 mM AgNO_3_ in the jacketed glass container. The mixture dispersion was stirred for 5 h with a magnetic bar at room temperature to allow the ion exchanges on the surfaces of the nanospheres to reach equilibrium. After that, 10 mL of 10 mM NaBH_4_ solution was added into the dispersion and the resulting mixture was allowed to react at 0 °C for 2 h while stirring. The suspension was centrifuged for 20 min at the rate of 12,000 r·min^−1^ and the precipitate was dispersed in 30 mL of distilled water. This centrifugation-redispersion cycle was repeated four times in order to remove the un-reacting species.

### 2.4. Characterization of PSA Nanospheres and nAg@PSA Nanocomposites

The morphologies and particle sizes of PSA nanospheres were characterized via scanning electron microscopy (SEM; Hitachi S4800, Hitachi Ltd., Tokyo, Japan). The appearance of the nanospheres were observed by scanning electron microscopy. The mean size of PSA was calculated by the software of nano measurer.

The nAg@PSA composite materials were investigated by transmission electron microscopy (TEM; JEOL JEM-2010, JEOL Ltd., Tokyo, Japan) to demonstrate the nanoparticle features, and the location of the silver particles on the latex surface.

The amount of carboxyl groups on the PSA latex particle was determined by titration with NaOH standard volumetric solution under monitoring of the conductivity of the suspension. From the turning point of the titration curve, we determined the number of the carboxyl moiety per particle [25].

Quantitative determination of the main elements on the PSA nanosphere surface was achieved by X-ray photoelectron spectroscopy (XPS; Thermo Fisher Scientific K-Alpha, Thermo Fisher Scientific, Waltham, MA, USA). Chemical bonding information was obtained from the PSA nanosphere surface by XPS analysis.

The crystalline structures of silver particles supported on the PSA nanosphere surface were characterized by X-ray diffraction (XRD; PANalytical X’pert Pro, PANalytical Co., Almelo, The Netherlands) and the XRD data was collected in a 2*θ* range of 30–80° at a scan rate of 4°/min.

Ultraviolet-visible (UV-vis; Shimadzu UV-2550, Shimadzu, Kyoto, Japan) spectroscopy was employed for qualitative characterization of the optical properties of silver nanoparticles supported on poly(styrene-*co*-acrylic acid) composite nanospheres. UV-vis absorption spectrum was obtained in the range from 300 to 900 nm. The distilled water was used as a blank solution.

The content of silver nanoparticle coatings on poly(styrene-*co*-acrylic acid) composite nanospheres was analyzed by thermogravimetric analysis (TG; Perkin Elmer Pyris 1, Perkin-Elmer Co., Norwalk, CT, USA). TG was carried out at a heating rate of 10 °C·min^−1^. Thermal behavior of nAg@PSA composite nanospheres provides quantitative results about Ag nanoparticles covered on composite nanospheres.

## 3. Results and Discussion

### 3.1. Effect of Different Factors on the Particle Size and Morphology of PSA Nanospheres

#### 3.1.1. Amount of Acrylic Acid

**Figure 2:**
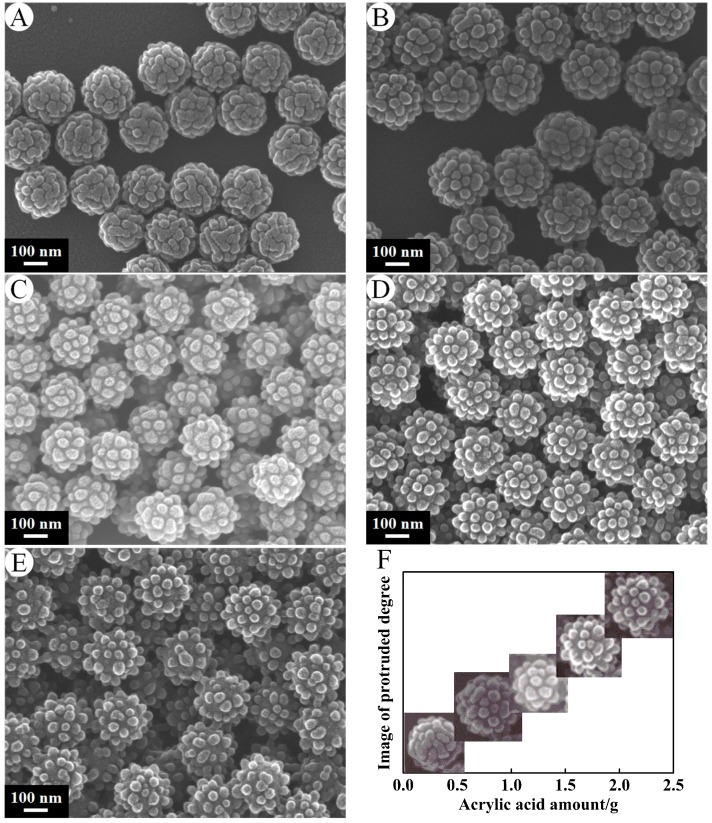
SEM micrographs of PSA nanospheres under preparation conditions of the acrylic acid (AA) amount (**A**) 0.6 g, (**B**) 1.2 g, (**C**) 1.5 g, (**D**) 1.8 g, (**E**) 2.1 g. Other conditions: St, 6 g; potassium persulfate (KPS), 0.12 g; stirrer speed, 300 r·min^−1^. Effects of AA content on the protruded degree of the PSA nanospheres (**F**).

**Figure 3:**
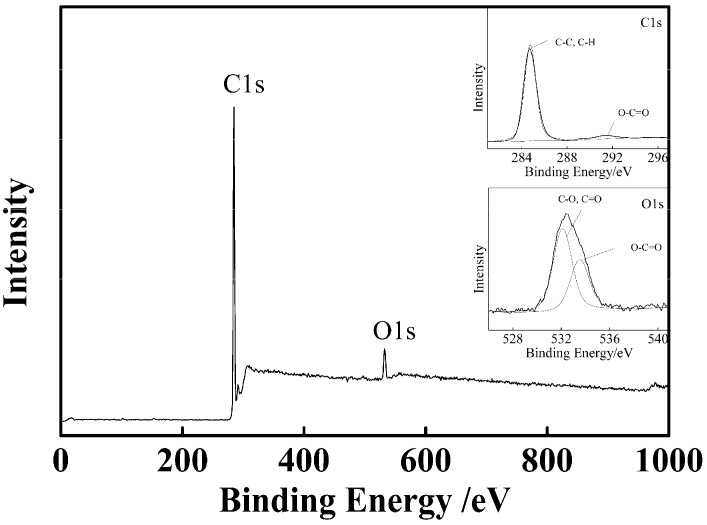
XPS spectrum of PSA0 nanospheres.

Figure 2 shows SEM micrographs of PSA nanospheres prepared with different amount of AA under a certain amount of St (6 g) and initiator (0.12 g) with stirring at the speed of 300 r·min^−1^. A–E correspond to PSA1–PSA5. The particle size distributions could be observed in Figure 2. The particles which were synthesized by soap-free emulsion polymerization have uniformly distributed granular diameters. The mean sizes of PSA were 220.48 ± 8.61 nm, 258.83 ± 9.48 nm, 220.46 ± 7.56 nm, 239.80 ± 9.11 nm, 238.36 ± 9.93 nm, respectively. The size of PSA nanospheres had significant changes with an increase of the AA amount (*p* = 5.19 × 10^−26^ < 0.05). These SEM micrographs also indicated that these nanospheres had interesting surface geometry. There were many half-spheres, which were uniformly distributed on the surface of the PSA nanospheres. Those half-spheres protruded more with increase of feeding amount of AA or the feed ratios of AA and St. The half-spheres became almost spherical when the mass of AA increased to 2.1 g. This is reasonable, since polyacrylic acid is more hydrophilic than polystyrene, more polyacrylic acid will accumulate and bulge into aqueous bulk solution with an increase of the amount of acrylic acid. XPS was conducted to further verify the elemental composition and chemical status of PSA nanospheres. The result was shown in Figure 3. The spectrum indicated the existence of carbon and oxygen (hydrogen cannot be detected by XPS). The C1s spectrum in the inset of Figure 3 revealed that it consists of two peaks arising from C–C/C–H groups and O–C=O groups. The O1s spectrum in the inset of Figure 3 can be fit to two peaks, which can be attributed to C–O/C=O groups and O–C=O groups, respectively. These data proved the presence of carboxyl groups on the surface of PSA nanospheres. This indicates that polyacrylic acid was distributed on the surface of PSA nanospheres.

#### 3.1.2. Amount of Initiator

**Figure 4:**
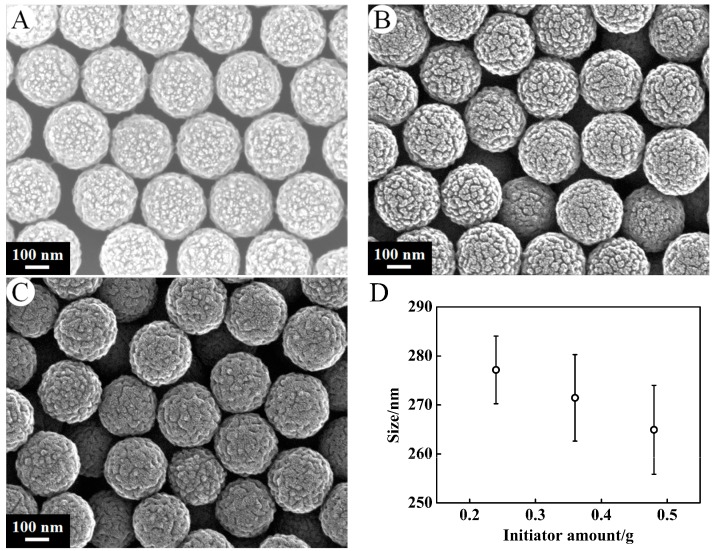
SEM micrographs of PSA nanospheres under preparation conditions of the initiator amount (**A**) 0.24 g, (**B**) 0.36 g, (**C**) 0.48 g. Other conditions: AA, 0.3 g; St, 6 g; stirrer speed, 300 r·min^−1^. Effects of initiator content on the size of the PSA nanospheres (**D**).

The micrographs of PSA nanospheres prepared with different initiator amount under a certain amount of St (6 g) and AA (0.3 g) with stirring at the speed of 300 r·min^−1^ were shown in Figure 4. A–C correspond to PSA6–PSA8. Each PSA spheres uniformly dispersed with average diameters of 277.16 ± 6.90 nm, 271.45 ± 8.83 nm, 264.93 ± 9.08 nm, respectively. It can be seen that the dosage of the initiator has effect on the size of polymer microsphere (*p* = 0.0001 < 0.05) and has no significant effect on the morphology.

#### 3.1.3. Monomer Concentration

**Figure 5:**
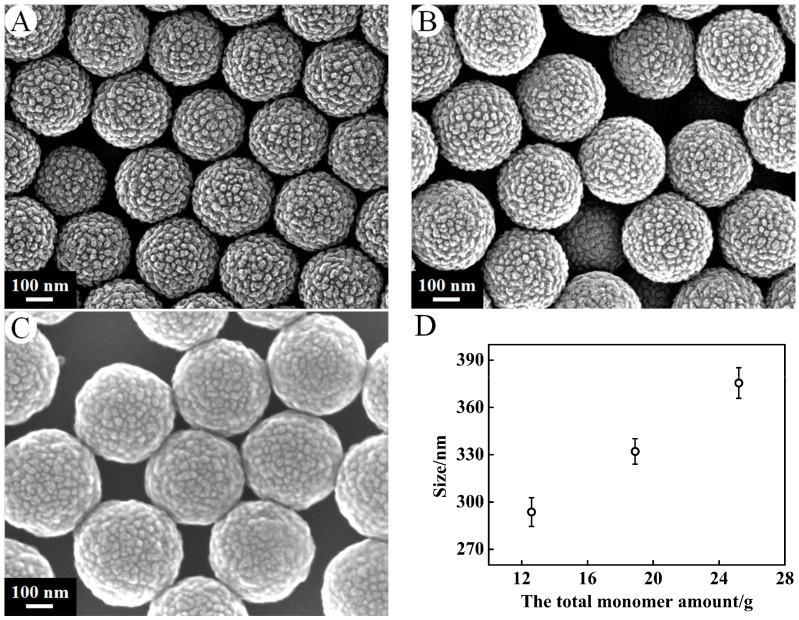
SEM micrographs of PSA nanospheres under preparation conditions of the total monomer amount (**A**) 12.6 g, (**B**) 18.9 g, (**C**) 25.2 g. Other conditions: KPS, 0.24 g; stirrer speed, 300 r·min^−1^. Effects of the total monomer amount on the size of the PSA nanospheres (**D**).

Figure 5 presents the typical SEM photographs of the PSA spheres synthesized by 0.24 g of KPS at the stirrer speed of 300 r·min^−1^ with varying amount of monomer from 12.6 g, 18.9 g to 25.2 g. The monomer ratio (styrene:acrylic acid) was 20:1 by weight. A–C correspond to PSA9–PSA11. It can be seen that the PSA spheres are all spherical in shape and almost monodispersed with mean sizes ranging from 293.63 ± 9.17 nm, 332.10 ± 8.05 nm to 375.54 ± 9.72 nm with the increase of monomer concentration. Individually stable particles were obtained at all concentrations of monomer. As monomer concentration increased, the reaction rate was speeded up, and then the larger particles were obtained in the same time. The surface morphology of PSA nanospheres did not change with the variation of the monomer concentration.

#### 3.1.4. Stirrer Speed

**Figure 6:**
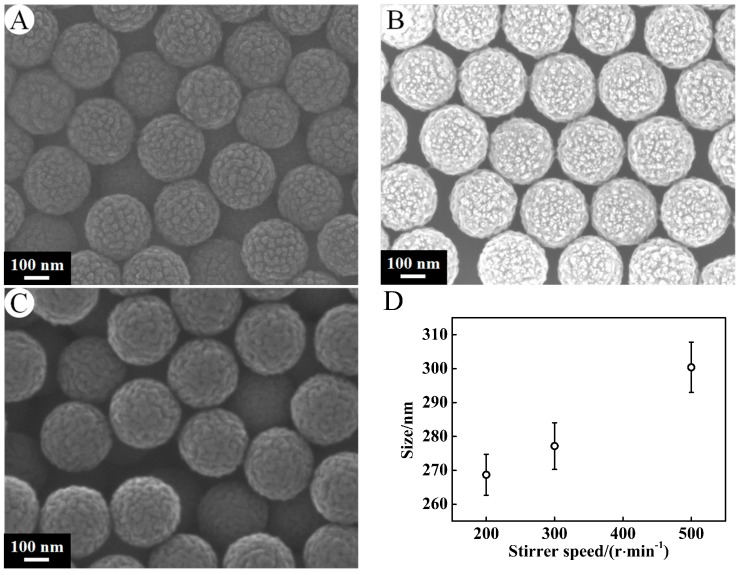
SEM micrographs of PSA nanospheres under preparation conditions of the stirrer speed (**A**) 200 r·min^−1^, (**B**) 300 r·min^−1^, (**C**) 500 r·min^−1^. Other conditions: AA, 0.3 g; St, 6 g; KPS, 0.24 g. Effects of stirrer speed on the size of the PSA nanospheres (**D**).

In Figure 6, the PSA nanospheres prepared by 0.3 g of AA, 6 g of St, 0.24 g of KPS with varying the stirrer speed from 200 r·min^−1^, 300 r·min^−1^ to 500 r·min^−1^ were shown. A, B, C correspond to PSA12, PSA6, PSA13. The average diameters of the polymer spheres rose from 268.69 ± 6.06 nm, 277.16 ± 6.90 nm to 300.39 ± 7.42 nm as the stirrer speed increased from 200 r·min^−1^, 300 r·min^−1^ to 500 r·min^−1^, respectively. With an increasing stirrer speed, the monomer is dispersed into monomer droplets, and the surface area of monomer droplets per unit volume solution is larger. Larger specific surface area causes more free radicals to adsorb onto the surface of monomer droplets and less effective free radicals for the formation of micelles. Therefore, this leads to a decrease in the number of polymer particles formed, and the size of the particle becomes larger.

### 3.2. Amount of Carboxyl in PSA Nanospheres

The density of PSA suspension was determined by drying and weighing a sample volume of PSA suspension. The sizes of the samples were examined by SEM and then the microsphere volume and surface area were calculated via the particle size. The volume was converted into the weight by use of the density of polystyrene (1.05 g·cm^−3^) on the assumption that the density of the poly(styrene-*co*-acrylic acid) was the same as that of polystyrene. Then the number of the nanospheres of a stock suspension was calculated.

**Figure 7:**
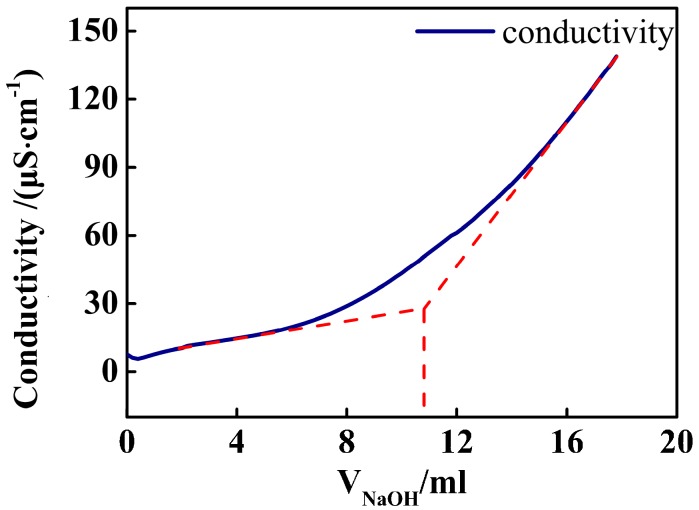
Conductometric titration curves of the PSA suspension, a case of PSA4.

Amount of carboxyl groups on the particle was determined by titration with NaOH under monitoring of the conductivity. The conductivity decreased initially with addition of NaOH because the molar conductivity of sodium ion is smaller than that of hydrogen ion. Further addition of NaOH over the equivalent point increased the conductivity, because of extra amounts of Na^+^ and OH^−^. From the turning point of the titration curve, we determined amount of carboxyl of the suspension, as was shown in Figure 7. The number of the carboxyl per particle was calculated by the carboxyl amount of the suspension and the number of the nanospheres.

**Figure 8:**
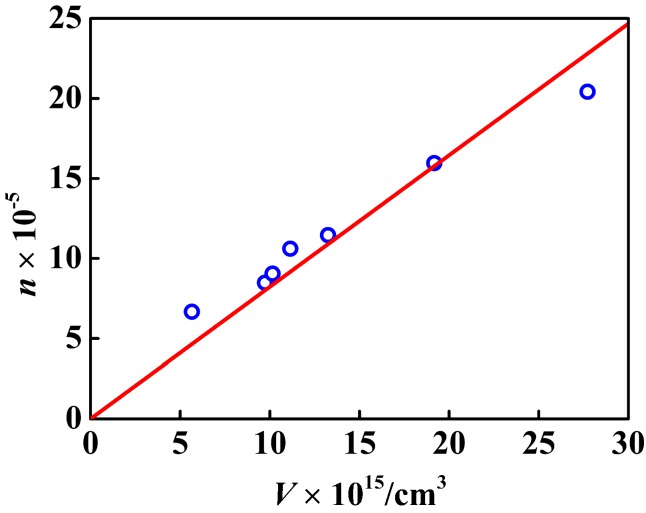
Variation of *n* (the number of the carboxyl moiety per PSA nanosphere) with *V* (the PSA nanosphere volume).

**Figure 9:**
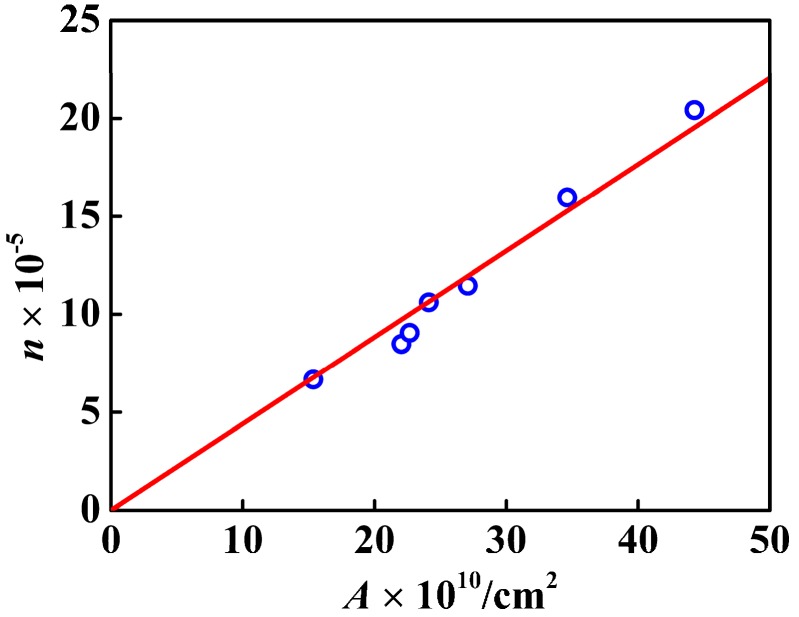
Variation of *n* (the number of the carboxyl moiety per PSA nanosphere) with *A* (the PSA nanosphere surface area).

**Figure 10:**
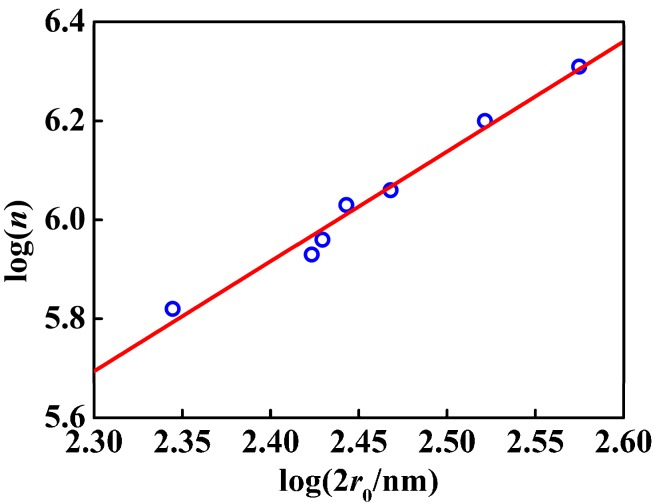
Logarithmic plots of *n* (the number of the carboxyl moiety per PSA nanosphere) against diameters of PSA nanospheres, 2*r*_0_.

**Figure 11:**
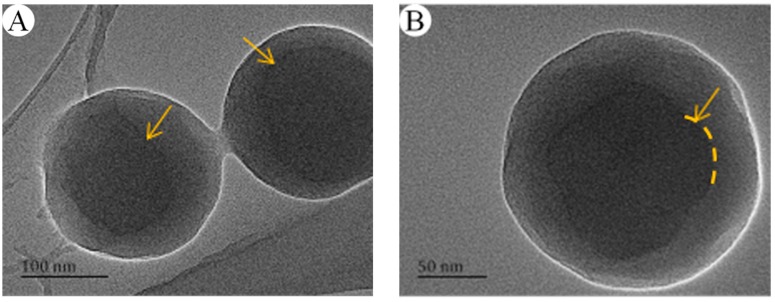
TEM images of the PSA0 nanospheres with different scale bars (**A**) 100 nm, (**B**) 50 nm.

From the linear fit in Figure 8 and Figure 9, not only the nanosphere surface area but also the volume had a linear relationship with the number of –COOH per particle. The correlation coefficient between the number of –COOH per particle and the volume was 0.9077, and that between the number of –COOH per particle and the nanosphere surface area was 0.9714. Compared with the linear correlation between the number of –COOH per particle and the volume, the linear correlation between the number of –COOH per particle and the nanosphere surface area was stronger. Figure 10 showed logarithmic variation of the number of the loaded carboxy moiety per particle against the diameters. The plot had a linear relation with the slope of 2.2, indicating that the carboxy would be mainly distributed in the spherical surface. PSA nanospheres showed definite boundaries between the core-shell structure and the thick shell, as indicated in Figure 11 by the yellow arrows in part A and the dashed line and yellow arrow in part B. From Figure 3, the presence of carboxyl groups on the surface of PSA nanospheres was proved. So, PSA is the styrene core/acrylic acid shell nanospheres, and carboxyl groups are distributed uniformly on the latex surface.

### 3.3. Effect of the Amount of Carboxyl on the Deposition of Ag Nanoparticles

**Figure 12:**
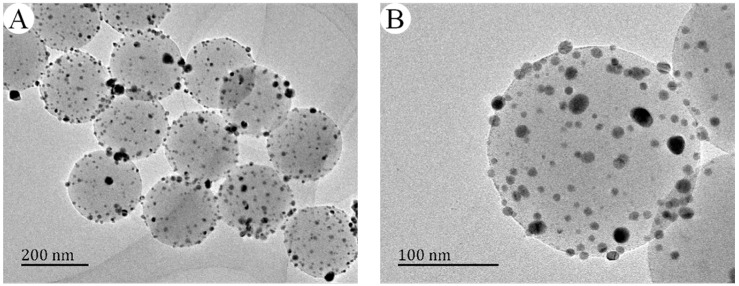

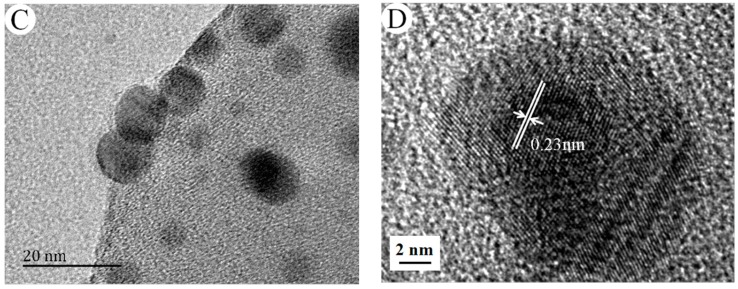
Representative TEM images of nAg@PSA composite nanospheres obtained from PSA0 nanospheres with different scale bars (**A**) 200 nm, (**B**) 100nm, (**C**) 20 nm, (**D**) 2 nm.

**Figure 13:**
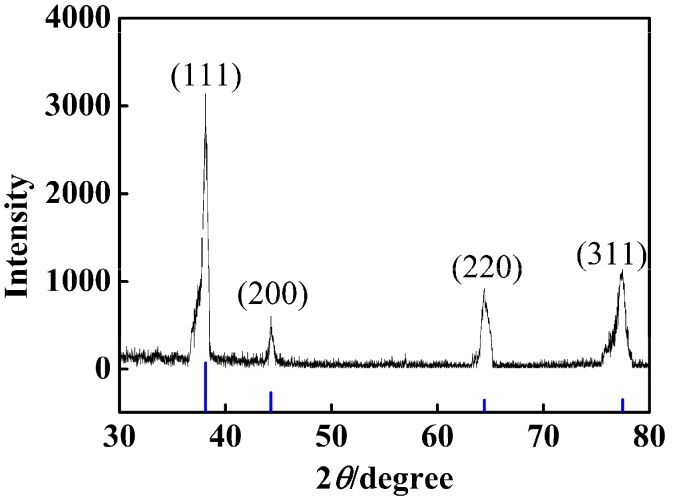
XRD of nAg@PSA composite nanospheres obtained from PSA0 nanospheres.

**Figure 14:**
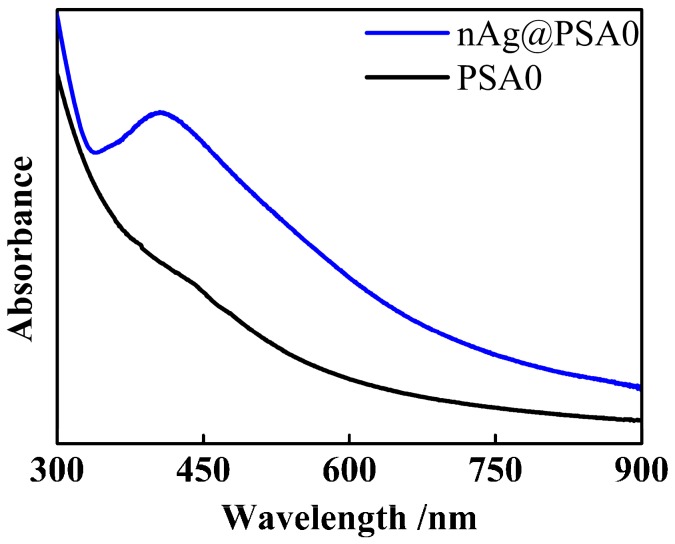
Absorption spectra of PSA0 nanospheres and nAg@PSA composite nanospheres obtained from PSA0 nanospheres.

Based on the method in the Experimental section, a series of nAg@PSA composite nanospheres were prepared using various PSA nanospheres while keeping all other parameters unvaried, and their TEM images are shown in Figure 12. No isolated Ag nanoparticles that have fallen off the polymer nanospheres are observed in it, which clearly show that the all the Ag NPs are firmly anchored on the polymer spheres. From Figure 12D, we can gain more insight into the microstructure of Ag nanoparticles. The lattice fringes of the silver nanoparticle with the lattice spacing of about 0.23 nm corresponded to the (111) plane of silver crystal, which confirmed that Ag was successfully deposited. Besides, the XRD pattern also provided evidence of the well-defined Ag crystallization. As shown in Figure 13, sharp peaks were observed at 2*θ* of 38.1°, 44.3°, 64.4°, and 77.5°, corresponding to diffractions from the (111), (200), (220), and (311) planes of face centered cubic (fcc) phase of Ag (JCPDS card No. 04-0783), respectively. Absorption spectra of PSA nanospheres and nAg@PSA composite nanospheres are shown in Figure 14. The absorption spectrum of nAg@PSA nanospheres shows a peak at 407 nm which corresponding to the surface plasmon absorption (SPR) of the nanosilver particles.

**Figure 15:**
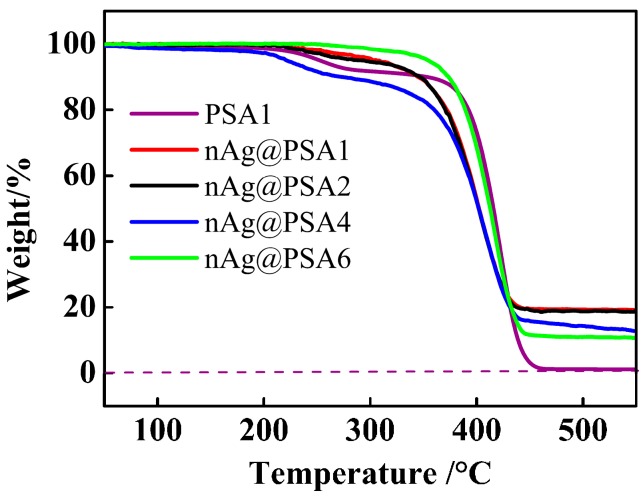
TG curves of PSA nanospheres and nAg@PSA composite nanospheres obtained from PSA1, PSA2, PSA4, PSA6 nanospheres.

**Figure 16:**
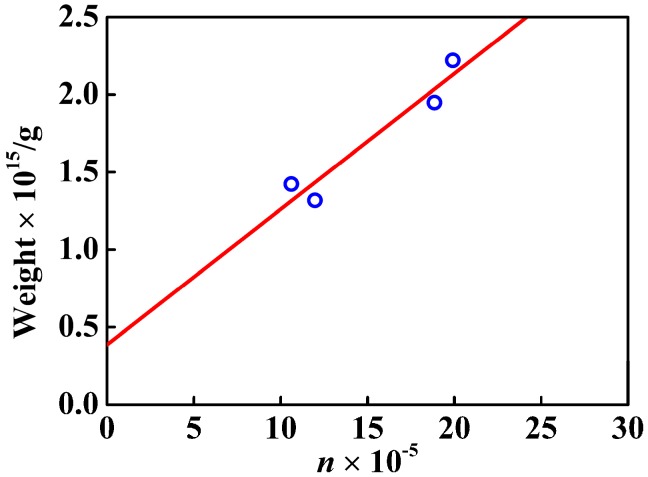
Variation of the Ag nanoparticles weight of composite nanospheres with the number of the carboxyl per particle, *n*.

Thermogravimetric analysis further provides quantitative results about Ag nanoparticles covered on composite nanospheres. TG curves of PSA nanospheres and nAg@PSA composite nanospheres were shown in Figure 15. The PSA spheres are almost completely degraded by 550 °C and the residual in the nAg@PSA particles after pyrolysis can be deemed to be the content of Ag in the particles. Then the silver nanoparticles weight of composite nanospheres was calculated. The increase of Ag weight on PSA spheres with increasing of carboxyl content can be demonstrated by TG data, and were also observed by Figure 16. This can be explained by the forming mechanism of silver nanoparticles on the surface of composite nanospheres. These results indicated that the presence of surface carboxyl groups facilitated attachment of silver ions onto the microsphere surfaces by electrostatic interaction, which allowed, upon reduction, on the microsphere surfaces, uniform initial formation of silver nuclei that served as seeds for further growth to form the observed Ag-NPs shells. So we can conclude that the number of the carboxyl on the surface of composite nanospheres is a significant factor to control the content of Ag nanoparticles supported on composite nanospheres.

## 4. Conclusions

In summary, we demonstrated that monodisperse poly(styrene-*co*-acrylic acid) (PSA) nanospheres were synthesized via soap-free emulsion polymerization and Ag nanoparticles on the PSA composite nanospheres were prepared in situ through controlled interfacial reduction. SEM .micrographs indicated that PSA nanospheres with narrow particle size distributions had interesting surface geometry. The particle size of PSA nanospheres was influenced by changes in the amount of acrylic acid and initiator. The increase in the monomer concentration and the stirrer speed respectively gives larger particles. A great many of half-sphere arrays were uniformly distributed on the surface of PSA nanospheres. Those half-spheres protruded more with the feeding amount of AA or the ratios of AA and St increasing. The surface morphology was not affected by the initiator amount, the monomer concentration, or the stirrer speed. The carboxyl groups per nanosphere had good linear relationship with the nanosphere surface area, and the carboxyl groups content of nanospheres was directly proportional to the nanosphere surface area. These results concluded that the carboxyl groups were mainly distributed on the surface of the PSA nanospheres consisting of styrene core and acrylic acid shell. As the content of carboxyl groups increased, more Ag nanoparticles were coated onto the surface of nanospheres, and the Ag nanoparticles content of composite nanospheres increased.

## Figures and Tables

**Table 1:** Recipes of poly(styrene-*co*-acrylic acid) (PSA) composite nanospheres.

Sample Code	AA/g	St/g	KPS/g	Stirrer Speed/r·min^−1^
PSA0	0.1	2	0.04	400
PSA1	0.6	6	0.12	300
PSA2	1.2	6	0.12	300
PSA3	1.5	6	0.12	300
PSA4	1.8	6	0.12	300
PSA5	2.1	6	0.12	300
PSA6	0.3	6	0.24	300
PSA7	0.3	6	0.36	300
PSA8	0.3	6	0.48	300
PSA9	0.6	12	0.24	300
PSA10	0.9	18	0.24	300
PSA11	1.2	24	0.24	300
PSA12	0.3	6	0.24	200
PSA13	0.3	6	0.24	500
